# Large-Scale Compatibilization of Postconsumer Polyolefins in the Presence of Paraffin Wax as a Rheology Modifier

**DOI:** 10.1021/acsomega.4c10910

**Published:** 2025-04-11

**Authors:** Anurag Ganapathi, Rishi Sharma, Mohamed A. Abdelwahab, Muhammad Rabnawaz

**Affiliations:** School of Packaging, Michigan State University, East Lansing, Michigan 48824-1223, United States

## Abstract

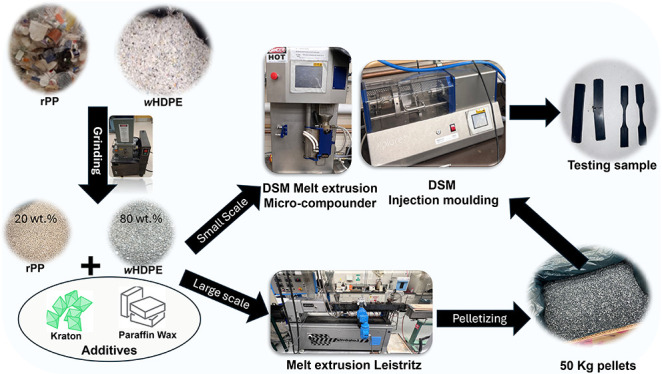

Postconsumer polyolefins (r-POs) are leading plastic waste contributors today. This study reports, for the first time, the compatibilization of r-POs at a 50 kilogram (kg) scale with a styrene block copolymer compatibilizer in the presence of paraffin waxes as rheology modifiers (RMs). The addition of the rheological modifier (RM) and compatibilizer enhances the melt flow indices (MFIs) and mechanical properties, respectively. One aspect of this study is to compare the effectiveness of low-cost paraffin wax to that of specialized and expensive RMs in r-POs. The mechanical and rheological properties such as the melt flow index (MFI) of r-POs were compared in the presence of two types of RMs. Next, the study explores the challenges encountered when scaling the compatibilization of r-POs in the presence of paraffin wax from a 10-g to a 50-kg scale. The mechanical properties were determined and compared for samples at different scales. The study further investigated the effect of the method used for blending paraffin wax with r-PO and its impact on the value of their MFI and mechanical properties. This at-scale validation could pave the way for the commercialization of r-POs.

## 1 Introduction

Plastics are widely used in numerous applications due to their lightweight, low cost, and ease of processing. As a result, plastic consumption has increased over the last few decades, surpassing 400 million tons per year. Packaging accounts for one-third of total plastic consumption, and nearly half of the plastic in municipal solid waste comes from packaging alone.^1^ This is because packaging often utilizes plastics for single use, resulting in a very short service life.^1^ Due to low recycling rates of plastic worldwide, especially in the United States, plastics are accumulating in landfills and the environment.^2^ When plastic leaks into the environment, it can degrade into tiny particles known as microplastics. These microplastics pose a risk to both the planet and our health, and recent reports reported that microplastics have been found in human blood and other parts of the body.^3,4^

Recycling is a crucial solution to mitigate plastic waste. Transitioning from an open-loop to a closed-loop plastic economy is necessary to preserve resources and reduce pollution. Mechanical recycling is currently more viable than chemical recycling due to its lower cost and mature processing methods.^5−7^ However, polyolefins (POs), which constitute nearly 50% of all plastics produced and more than two-thirds of packaging plastics, present significant challenges in recycling.^8^ Their stable carbon–carbon bonds make chemical recycling difficult, leaving mechanical recycling as the primary approach.^9^

However, there are two strong barriers to the mechanical recycling of POs (r-POs). One barrier is that r-POs are very difficult to separate from each other.^10^ When POs, which are lighter than water, are separated from other plastic waste by the sink-float method, r-POs rise to the surface of the water. However, separating r-POs like high-density polyethylene (HDPE), low-density polyethylene (LDPE), linear low-density polyethylene (LLDPE), and polypropylene (PP) from each other is costly. Thus, the cost-effective way to process r-POs is to avoid sorting them into individual types of plastics.^10^ However, this creates a substantial challenge due to the significant variations that exist in the melt flow indices (MFIs) of r-POs. Achieving a well-defined MFI is vital in controlling the processing of applications that make anything out of plastics. Still, the MFIs of postconsumer POs vary from batch to batch, and this variation becomes a significant problem. The second barrier is the need for an inexpensive way to compatibilize r-POs using a low amount of compatibilizers.^11−13^

The first barrier can be addressed by using rheology modifiers (RMs) to control the melt flow of mixed r-POs. Rabnawaz and co-workers^13^ recently reported that RMs such as Sol B52 can be used to control melt flow.^13^ They also derived master curves and model equations to predict and tailor the MFIs of mixed POs by using a model equation. However, that study focused on using expensive, specialized, chemically modified waxes. In contrast, this study aims to explore inexpensive, unmodified paraffin waxes and compare their effects on the rheology of r-POs with those of specialized waxes.

The second barrier in r-PO recycling is their low mechanical properties due to poor interfacial adhesion between different polymers of the r-POs. This issue is traditionally addressed by using compatibilizers.^14^ Compatibilizers improve interfacial adhesion between different polymers, thus enhancing their mechanical properties.^13,15^ Compatibilizers can be produced by reactive extrusion (in situ compatibilization) or by adding copolymers/compatibilizers. While both approaches have been well-explored for the compatibilization of r-POs, compatibilization is seldom explored in the presence of RMs. Rabnawaz and co-workers^13^ reported the effective use of compatibilizers, both in situ made and premade, to compatibilize r-POs in the presence of RMs. However, that study was conducted on a relatively small scale (2 kg) and used specialized, modified waxes rather than low-cost paraffin wax (PW).^13^

This study aims to test the compatibilization of r-POs at a larger scale using paraffin waxes as RMs. The purpose of RM addition was to tailor the MFI properties of r-POs and evaluate the mechanical properties of the recycled material to simulate the actual situation. Large-scale experiments were conducted at a 50 kg scale using paraffin wax as an RM, and the results were compared with smaller-scale tests (10 g, 1 kg, and 5 kg) to assess scalability and cost-effectiveness compared to specialty-modified waxes like Sasol B52. This scale is significant because it reflects the industrial-level processing required for practical applications, ensuring the findings can be feasibly implemented in commercial recycling operations.

## 2 Experimental Section

### 2.1 Materials

Table 1 shows the materials used in this study. The waste high-density polyethylene (wHDPE) plastics were obtained from PADNOS as mixed material and were density-separated by the sink-float method described in our previous article.^13^ DSC analysis showed that the plastic obtained from PADNOS was mainly wHDPE. The recycled PP (rPP) was sourced from waste plastic packaging (bottles, containers, and so forth) collected at the MSU surplus store in East Lansing, MI, USA. The waste material obtained from the surplus store was washed with detergent and rinsed with water. All impurities, labels, and other contaminants were removed, and then it was vacuum-dried overnight at 80 °C. The waste material was converted into small granules with the help of a granulator at the MSU School of Packaging (East Lansing, MI, USA) to obtain rPP granules. Two types of rheological modifiers (RMs) were used in the experiment. The first RM is a carboxy polymethylene resin (Sasol wax B52), produced by Sasol Performance Chemical (South Africa). It is pale yellow and formed using the Fischer–Tropsch process, which reacts with hydrogen and carbon monoxide to form unsaturated and unbranched molecules. This synthetic hydrocarbon’s linear structure leads to low product viscosity. It has a melting point of 58 °C and typically has a narrower carbon range compared to natural paraffin wax, and the molecules are usually more uniform in size due to the synthetic nature of the product.

**Table 1:** Summary of the Materials Used in This Study along with Their Material Codes and Purposes

material	code	purpose
waste HDPE	wHDPE	polymer
recycled polypropylene (MSU surplus)	rPP	polymer
Kraton	K	compatibilizer
Sasol B52-96-1001	B52	rheology modifier
paraffin wax	PW	rheology modifier

The second type of RM is paraffin wax (PW), procured from Sigma-Aldrich, which has a melting point of 60 ± 2 °C. Paraffin wax is a white, odorless solid saturated hydrocarbon with a chain length of C20 to C40.

Sasol B52 is typically more expensive than natural paraffin wax due to its complex and energy-intensive Fischer–Tropsch synthesis process and its controlled production nature.

Kraton (K) (Circular + C2000, Kraton, Houston, TX, USA), made of polystyrene and poly(ethylene-*co*-butylene), acts as a compatibilizer to enhance the mechanical properties of the recycled plastic used as compatibilizers. The polystyrene blocks are hard and stiff and can be thermally treated. The styrene concentration can range from 30 to 50% by weight. The butadiene percentage can range from 50 to 70% by weight.

### 2.2 Sample Preparation

The rPP surplus and wHDPE granules were dried at 80 °C to remove residual water before processing. The rPP and wHDPE granules were made into pellet form using a twin-screw extruder (Leistritz Extrusion machine, Michigan, USA). The rPP and wHDPE were mixed manually at different weight ratios (Table 2) at 220 °C and 100 rpm. After these polymers were mixed at the desired ratio, the mixture was combined with the different RMs (Sasol or paraffin wax) at various ratios and with a compatibilizer (+C2000) via a reactive extrusion process that was performed with a Leistritz extruder.

**Table 2:** Screening Process To Select Optimum PO Blends

sample	wHDPE (wt %)	rPP (wt %)
wHDPE	100	
rPP		100
80PO (m-POs)^a^	80	20
60PO^a^	60	40
40PO^a^	40	60
20PO^a^	20	80

a Number before PO denotes wt % of wHPDE in the PO blend.

An initial screening process was performed to select the optimum r-PO blend, which then underwent testing with the compatibilizer and RM to obtain mechanical properties and MFIs comparable to those of virgin HDPE.

The different pellet mixtures were microextruded in a twin-screw extruder (DSM Xplore 15 cm^3^) at 220 °C at 100 rpm for 2 min in the microextruder. The molten mixture was injected and molded to create impact bars and T-bones for tensile property measurements, respectively, as per the ASTMD256 and ASTMD638 protocols.

### 2.3 Characterization

Tensile and notched Izod impact tests were performed using impact bars and T-Bone specimens and kept for 48 h at room temperature before testing. The notched impact tests followed the ASTM D256 protocol using Ray Run test equipment with a 5.417 J hammer pendulum. The samples were initially notched with a TMI 22-05 notch cutter, ensuring that the notch size met the standard specifications. An Instron model 5565 (MA) tensile testing instrument was used to evaluate the tensile properties of the samples, following the ASTM D638-14 (type V) protocol, with a testing speed of 10 mm/min. The reported mechanical data represent the average of five measurements.

The Melt Flow Indices (MFIs) were measured using a Ray Run Melt Flow Index machine. Each m-PO, wHDPE, and rPP blend weighed 7–10 g, and the sample was heated at 230 °C using a 2.16 kg load to plunge the molten polymer from the barrel using the ASTM D1238 testing standard. A timed extrudate was gathered and weighed, with the melt flow rate calculated in grams per 10 min.

Differential scanning calorimetry (DSC) was performed with a DSC2500 Analyzer (TA Instruments, DE, USA). 5–10 mg of m-POs, wHDPE, and rPP samples were tested under nitrogen at a heating rate of 10 °C min^–1^. The samples were equilibrated at −80 °C and then heated to 220 °C. The cooling cycle and second heating cycle were used for data acquisition. The crystallinity was calculated using the formula *X*_c_ = [(Δ*H*_m_)/((1 – Ø) Δ*H*_m_*)]100, where Ø is the weight fraction of rPP or wHDPE in (m-POs) blends, and Δ*H*_m1_* and Δ*H*_m2_ are the predicted melting enthalpy of 100% for crystalline HDPE (293 J/g) and PP (207 J/g), respectively.^16,17^

SEM (scanning electron microscopy) was performed using a JEOL 6610LV, JEOL Ltd., Tokyo, Japan (tungsten hairpin emitter). m-POs, wHDPE, and rPP samples were frozen in liquid nitrogen and fractured with the help of a hammer and chisel. Then, these fractured samples were mounted on aluminum stubs using high-vacuum carbon tabs (SPI Supplies, West Chester, PA). The samples were coated with a gold thin-layer coating (≈30 nm thickness) in an Emscope Sputter Coater model SC 500 (Ashford, Kent, England) purged with argon gas.

## 3 Results and Discussion

### 3.1 Effect of PO Blends Composition on MFI and Mechanical Properties

**Figure 1:**
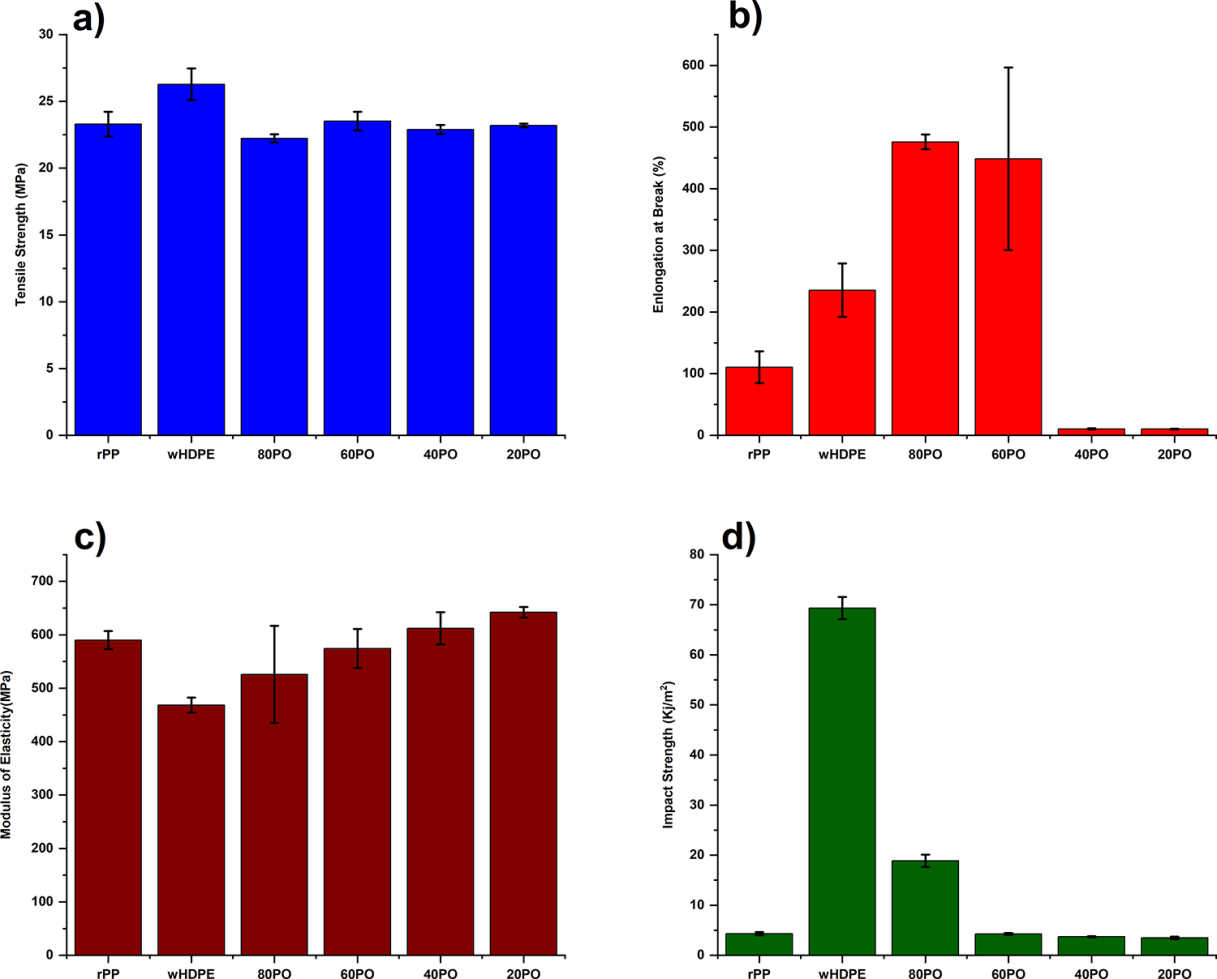
Mechanical properties of wHDPE, rPP, and PO blends. (a) Tensile Strength (MPa), (b) elongation at break (%), (c) modulus of elasticity (MPa), and (d) impact strength (kJ/m^2^).

Figure 1 depicts the mechanical properties of wHDPE, rPP, and PO blends. As shown in Figure 1a, the tensile strength of wHDPE is 26.2 MPa, and that of 40PO is 23.5 MPa, which indicates that the mixing of rPP with HDPE does not significantly impact the material’s tensile strength. However, the addition of rPP to wHDPE significantly impacts the elongation at break. wHDPE has a 235.4% elongation at break; after the addition of 20 and 40 wt % rPP, it increases to 476 and 449%, respectively. Increasing the amount of rPP in the blend decreases the percentage of elongation. For example, as shown in Figure 1b, with the addition of 60 and 80 wt % of rPP to wHDPE, the percentage of elongation decreased to 10.2 and 10.1%, respectively. The reduction in elongation percentage is due to the improper mixing of the polymer blend without any compatibilizer or RMs at 180 °C.^18−20^ One explanation for this issue is the phase separation of wHDPE, which produces wHDPE particles that act as nucleating agents for rPP. As a result, rPP’s spherulite size decreases, impeding its crystallinity. Blending PP and HDPE often reduces the overall crystallinity because the polymers have different crystallization rates and structures. This can lead to a more amorphous structure in the blend, especially in the interfacial regions where the polymers meet; decreased crystallinity in the blend may lead to lower tensile strength and an increase in the elongation percentage in comparison with the individual polymers, which can be observed in Figure 1a,b. These results are coherent with prior research suggesting that polyolefin blends display phase separation due to the limited compatibility between polyethylene (PE) and polypropylene.^11−13^ When the crystallinity of rPP is low, the tensile modulus is reduced, as shown in Figure 1c. When an impact force is applied, stress concentration and plastic deformation in the particles help absorb impact energy, thus enhancing the impact strength of r-PO blends.^21^ However, increasing the rPP content in recycled wHDPE reduces the impact strength significantly from 69.35 to 18.87 kJ/m^2^ with an rPP content of just 20 wt %.^19^ These observations are consistent with prior studies showing that increasing PP fractions in HDPE/PP blends leads to a stiffer material with lower impact strength.^19,20^

**Figure 2:**
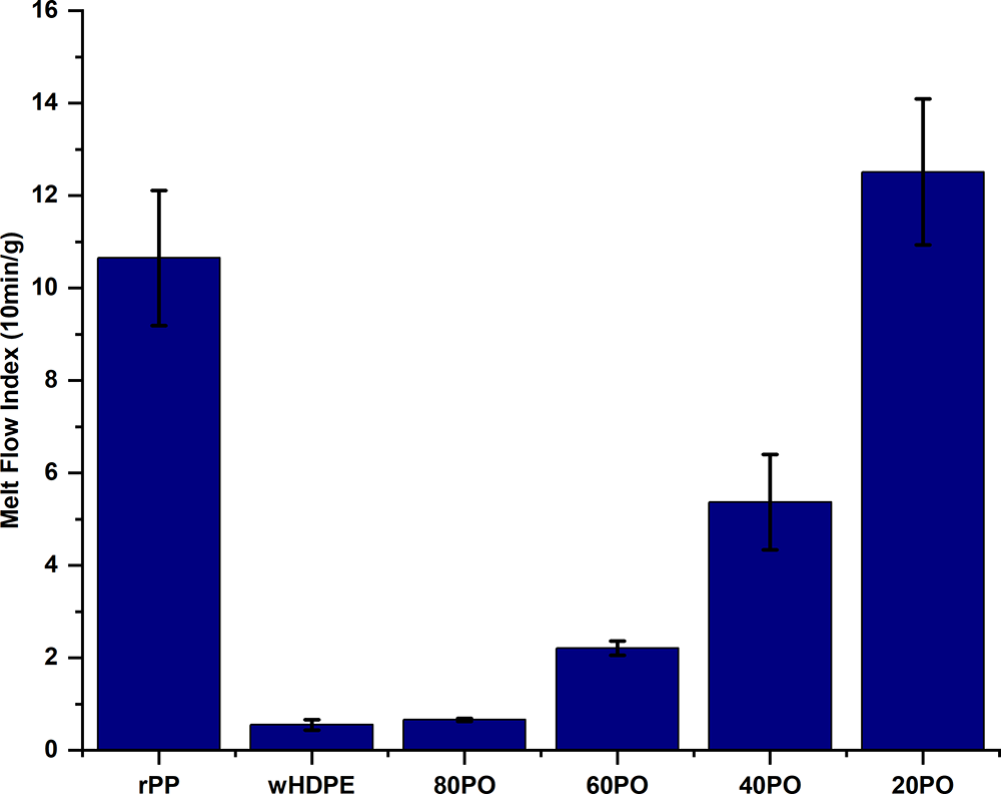
MFIs of wHDPE, rPP, and PO blends.

Similar trends were observed with the MFI data (Figure 2). Adding a higher wt % of rPP to the wHDPE polymer yielded a higher MFI. For example, the MFI of wHDPE was around 0.6 g/10 min, which increased to 1.3 and 2.2 g/10 min for blends containing 20 and 40 wt % rPP, respectively.^22^

### 3.2 Impacts of Sasol versus Paraffin Wax on the MFIs of PO Blends

**Figure 3:**
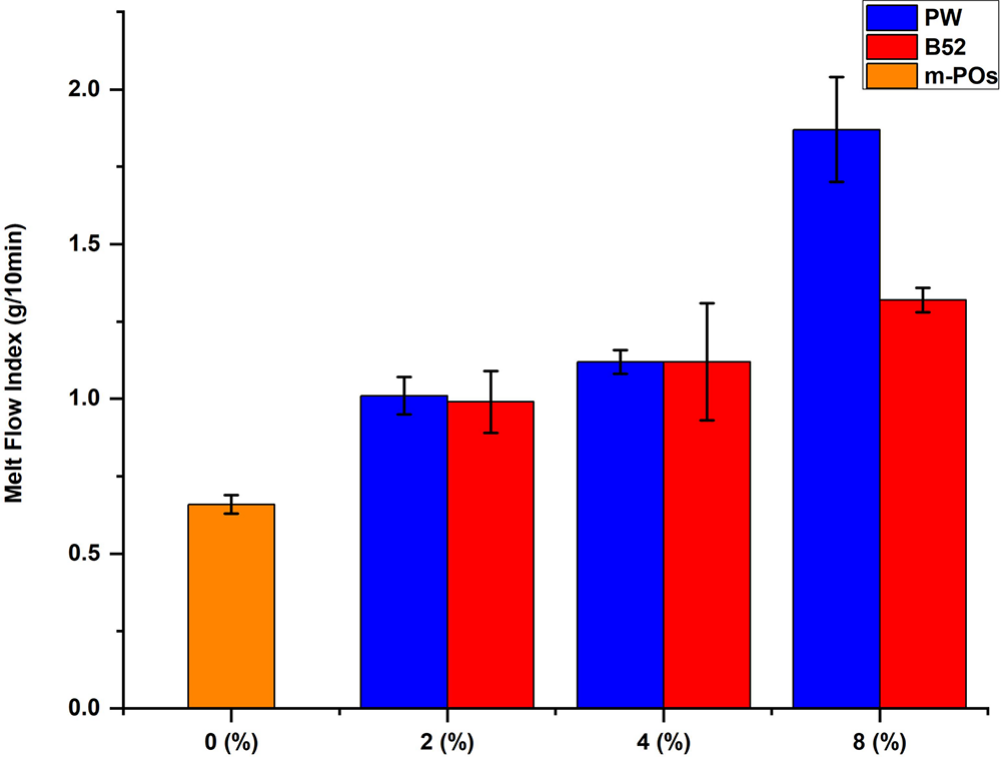
MFI comparison for wHDPE/20%rPP (m-POs) with and without PW and Sasol B-52 (B52).

An RM was added to the optimized wHDPE/20%rPP (m-POs) blend to achieve an MFI between 1 and 1.5 g/10 min, similar to that of virgin HDPE.^22^Figure 3 demonstrates the effect of Sasol (B52) and paraffin wax (PW) on the MFIs of the m-POs. The two systems of PW and B52 were compared at ratios of 2, 4, and 8 wt %. According to Figure 3, there was not much difference in the MFIs of the blends containing PW and those containing B52 at 2 and 4% ratios, respectively. However, there was a significant difference in the MFIs of the blends with a compatibilizer ratio of 8%, as the MFI was 1.9 g/10 min for the PW-containing system and 1.3 g/10 min for the B52-containing blend. This means that PW is more effective at improving flow properties at higher concentrations because its wax has a lower molecular weight.^13^ With the addition of 2% of PW and B52, the MFI was observed to be 1.0 and 0.9 g/10 min, respectively. Similarly, the MFI was observed to be nearly identical at around 1.1 g/10 min with the addition of 4 wt % of PW and B52 wax. These findings are consistent with previous studies that proved waxes as efficient MFI adjusters in polyolefin recycling.^23,24^

### 3.3 Impacts of Sasol versus Paraffin Wax on the Mechanical Properties of PO Blends

**Figure 4:**
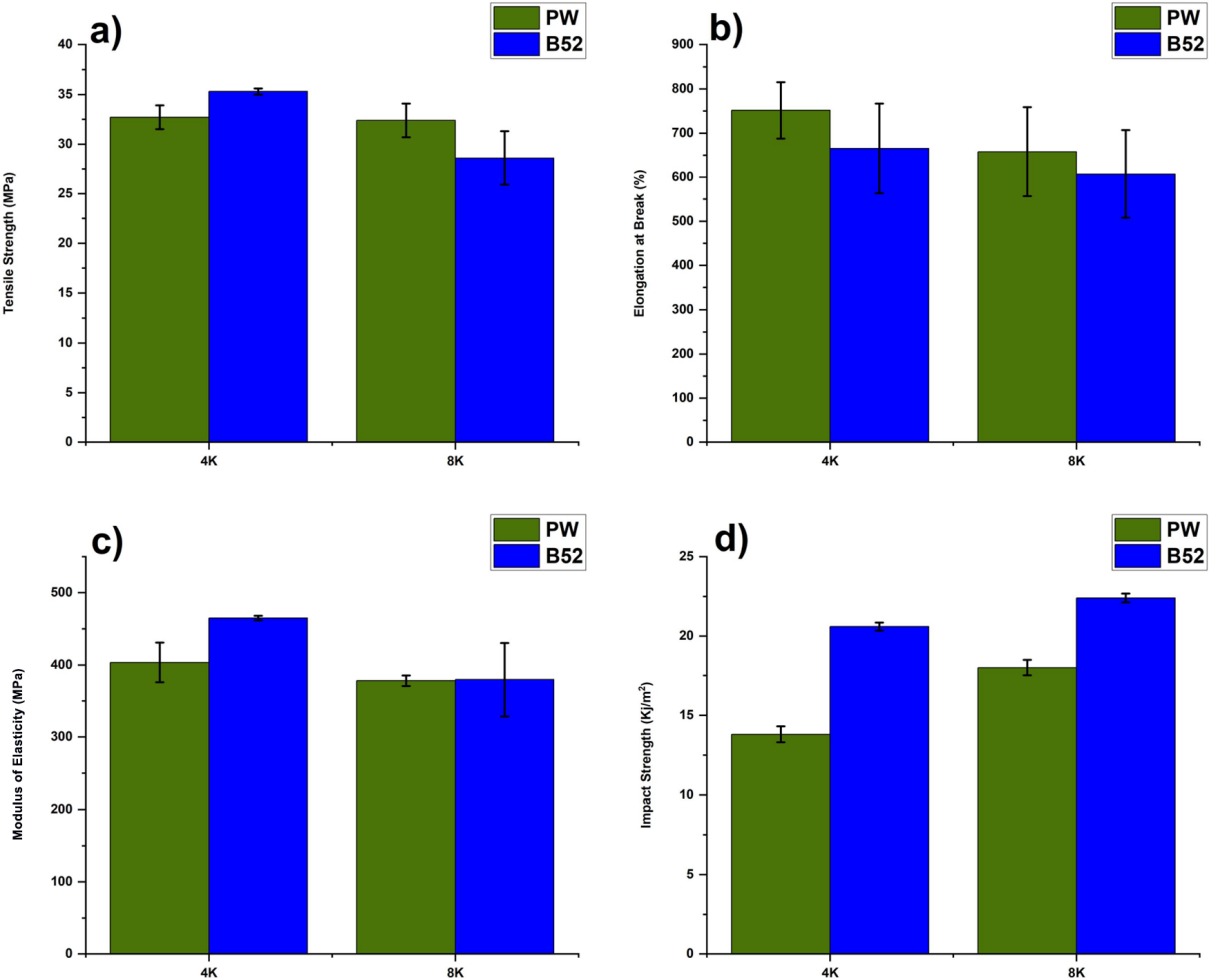
Mechanical characteristics of m-POs treated with PW and B52. (a) Tensile strength, (b) elongation at break, (c) modulus of elasticity, and (d) notched Izod impact strength. Comparative mechanical properties of m-POs treated with PW and B52. All samples have a wHDPE/20%rPP ratio (m-POs). Meanwhile, 4K and 8K denote samples with Kraton contents of 4 and 8 wt %, respectively.

The mechanical properties of the m-POs (wHDPE/20%rPP) blend with two different types of wax (B52 and PW) were assessed to see the difference between both systems. Kraton (K) was added as a compatibilizer to the blend to enhance the compatibility between wHDPE and rPP. Two compositions, 4 and 8% K with 5% wax, were added to the m-POs ratio. As observed in Figure 3, the MFI was between 1.9 g/10 min for a K content of 8 wt % and 1.1 g/10 min for a K content of 4 wt %, and to achieve an MFI of 1.5 g/10 min (like that of virgin HDPE), 5 wt % wax was chosen. By comparing Figures 1 and 4, it is evident that the mechanical properties, such as the elongation at break and modulus of elasticity, increase by ∼50% when the RMs PW and B52 are added into the m-POs blend. According to Puyvelde et al. (2001)^14^ and Muñoz et al. (2002),^15^ Kraton reduces interfacial tension and promotes adhesion between rPP and wHDPE phases, contributing to compatibilization. There was an increase in the modulus of elasticity found after RM addition. Prior findings suggest that compatibilizers improve compatibility and thus enhance their mechanical performance.^18,25^ Also, we can observe that the impact strength of the B52 system is slightly greater than that of PW. This may be due to the proper mixing of the m-POs blends in the presence of a rheology modifier, which resulted in reducing early interface cracking. As a result, elongation at break and modulus of elasticity increased by allowing uniform stress distribution inside the polymer matrix. Furthermore, rheology modifiers like B52 or PW help maintain cohesion between the compatibilizer and the m-POs blend.^13,15,23,24^

### 3.4 Effect of Scaling Up the Process

**Figure 5:**
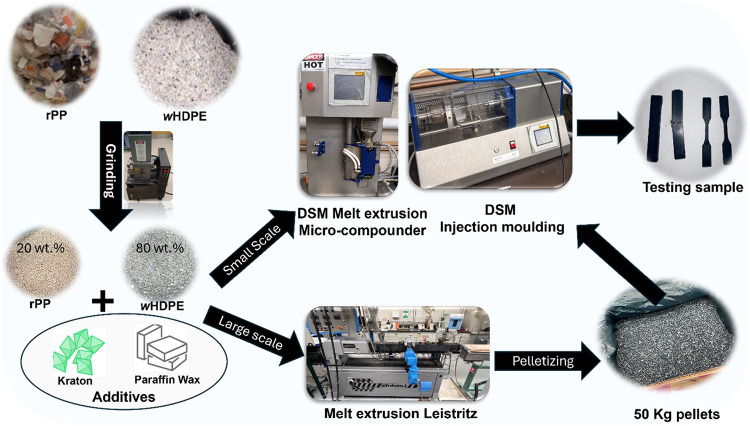
Pilot-scale testing of m-POs compatibilization involved several steps, including waste plastics collection and grinding, then small- and large-scale processing in DSM and Leistritz to produce the tensile and impact testing samples. (Photograph courtesy of M. Abdelwahab. Copyright 2025.)

A step-by-step approach for the pilot-scale testing is shown in Figure 5. This involved waste plastic collection, grinding waste plastic, and density separation of m-POs by the sink-float method. Finally, the m-POs were stabilized with Kraton in the presence of wax as an RM. Scaling up the process from a small scale (10 g) to the pilot scale/large scale (50 kg) was conducted in a gradual manner. Each time the scale was increased five- to 10-fold, the amount of Kraton required adjustment due to poor mixing of the rheology-modified m-POs. To address this issue, we developed a melt mixing method for the RM and m-POs to create a master batch of m-POs with a large amount of RM, which was then blended with m-POs. Once the melt-mixing step was developed for RM mixing with m-POs, the scalability became easier and more predictable, which led to the successful production of m-POs at a 50 kg scale.

**Figure 6:**
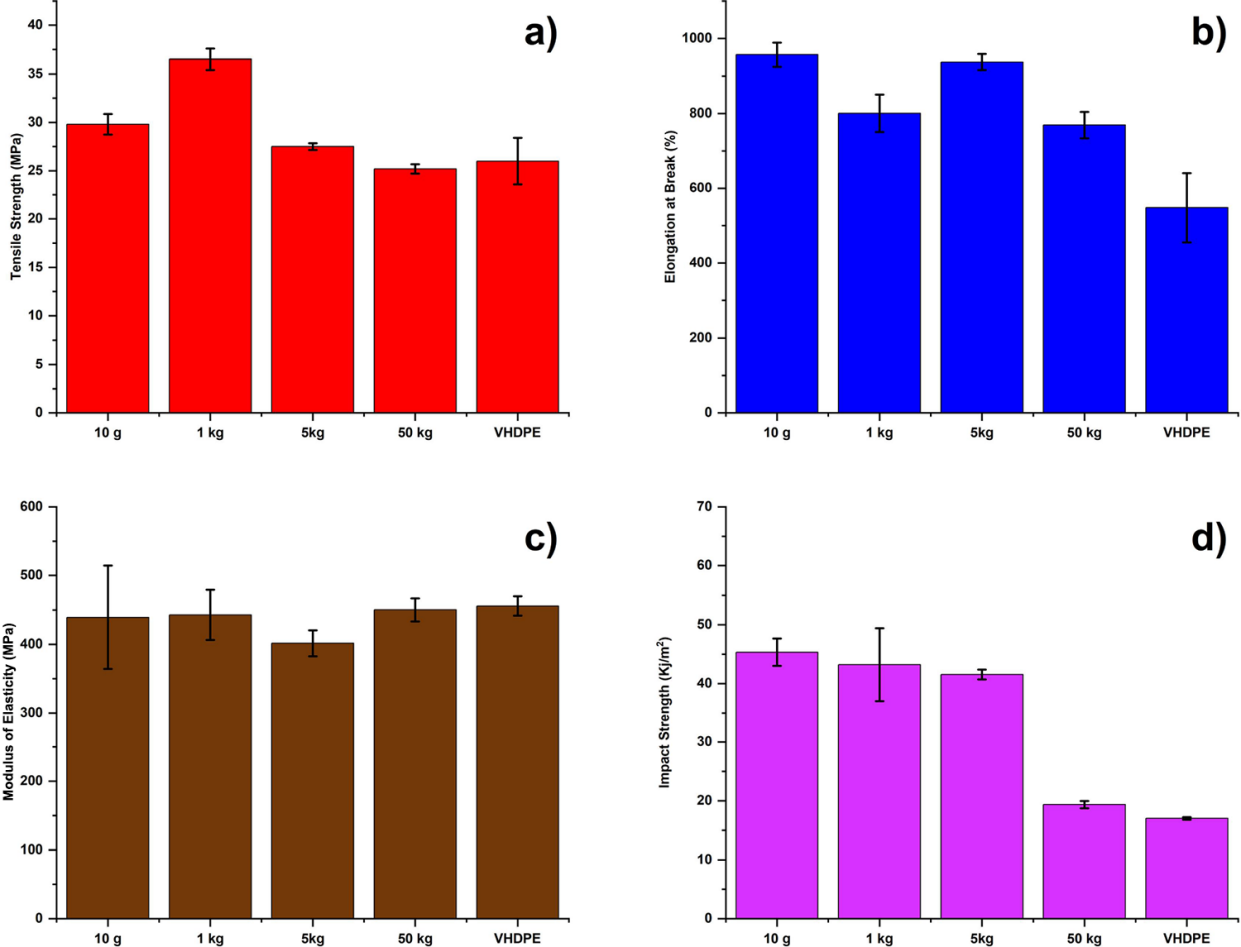
Mechanical properties of m-POs samples produced at various scales ranging from 10 g to 50 kg with 2.5 wt % of PW and 2 wt % K. These tensile properties include: (a) tensile strength (in MPa), (b) elongation at break (in percentage), (c) modulus of elasticity (MPa), and (d) impact strength (in kJ/m^2^).

Figure 6 exhibits the effects of scaling up the process from 10 g to 50 kg in a progression from 1, 5, and 50 kg production. The optimum system was tested, and the process was scaled up with an 80PO with 2.5 wt % PW and 2 wt % Kraton (K) in the composition. The reason for using 2.5% PW and 2% K was due to the scaling up of the process from a micro-DSM to a larger extruder such as the Leistritz system. Scaling up the process from a micro-DSM to a larger extruder (such as the Leistritz extruder) affects the viscosity profile, high shear and stress of the large-scale machine, better mixing capacity of the extruder, screw profile of the machine for better mixing flow rate, and heat transfer capacity of the mixture, which directly affect the mechanical properties of the m-POs blends, as the tensile strength of the polymer decreases with increasing the scale of the process. Figure 6 shows that tensile strength and impact strength decreased with the increasing scale of the process, due to extended heat exposure and shear stress during large-scale extrusion, resulting in minimal thermal deterioration.^26^ However, at larger scales such as 1 and 50 kg, more uniform dispersion is observed in the samples. These findings are consistent with earlier studies demonstrating that scale-up optimization is crucial to avoid property loss during large-scale polymer recycling.^9,27,28^ Also, the modulus of elasticity decreases due to the prolonged time required for the pellets to become mixed in the lengthy extruder. Similarly, the impact strength also decreases with the scaling up of the process due to the prolonged time required for the pellets to become mixed in the lengthy extruder. Scaling up the process decreases the tensile and impact strength of the polymer due to prolonged heat exposure. In particular, scaling up the extrusion process can change polymer rheology by altering the flow and heat transfer conditions, resulting in products with various properties. The purpose of scaling up is to reduce the discrepancies in performance requirements between the reference and target extruders; thereby, the best results were achieved at a 50 kg production.^26^ The scale of the process did not significantly impact the modulus.

### 3.5 Thermal Characterization

**Figure 7:**
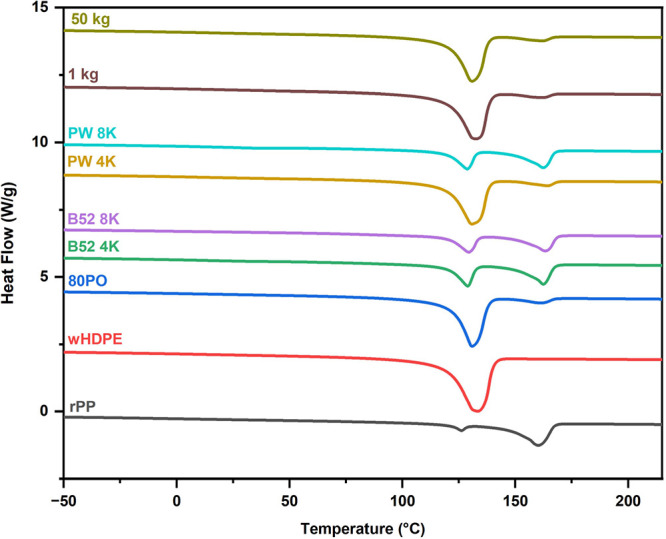
DSC of 2nd heating cycle for rPP, wHDPE, and m-POs samples.

Two separate melting peaks (*T*_m1_ and *T*_m2_), which represent the crystalline phases of wHDPE and rPP, respectively, were observed, as shown in Table S2 and Figure 7. This demonstrates that these two polymers are immiscible and that distinct crystalline domains exist.^29^

The melting temperatures (*T*_m1_ and *T*_m2_) of wHDPE and rPP are relatively consistent among samples tested at different scales. The cold crystallization temperature (*T*_c_) is greater in compatibilized samples (B52 4K, PW 4K), implying that the compatibilizer favors early nucleation during cooling. Despite the potential larger thermal degradation and more extended residence periods during extrusion on a larger scale, the thermal behavior remained essentially similar, demonstrating a successful scale-up with minimal thermal damage.^27,28^ The twin melting peaks slightly reduced using the Kraton compatibilizer, suggesting improved miscibility, yet with two distinct phases.^13,25^

The degree of crystallinity (*X*_c_) of the wHDPE/rPP blends varies depending on the composition and processing conditions. For the 80PO (80 wt % wHDPE and 20 wt % rPP), the crystallinity of both wHDPE and rPP phases is lower than that of the individual components, indicating a reduction in overall crystallinity caused by the disruption of the polymer chains at the interface. This is a common effect in incompatible polymer blends where phase separation inhibits regular crystalline packing. The crystallinity shifts even more when compatibilizer K (Kraton) and rheology modifiers (B52 or PW) are added. Samples containing 4% Kraton (B52 4K, PW 4K) exhibit a significant decrease in the crystallinity of the HDPE phase (*X*_c1_), while the crystallinity of the PP phase (*X*_c2_) increases. This implies enhanced interfacial adhesion, assisted by the compatibilizer, as previously reported for the HDPE/PP system.^30,31^ Samples prepared at larger scales (1 and 50 kg) had lower crystallinity (*X*_c1_ and *X*_c2_) than those of small-scale samples, particularly for wHDPE. This is most likely owing to higher heat degradation and longer residence periods during extrusion on a larger scale.^32,33^

### 3.6 Morphological Characterization

**Figure 8:**
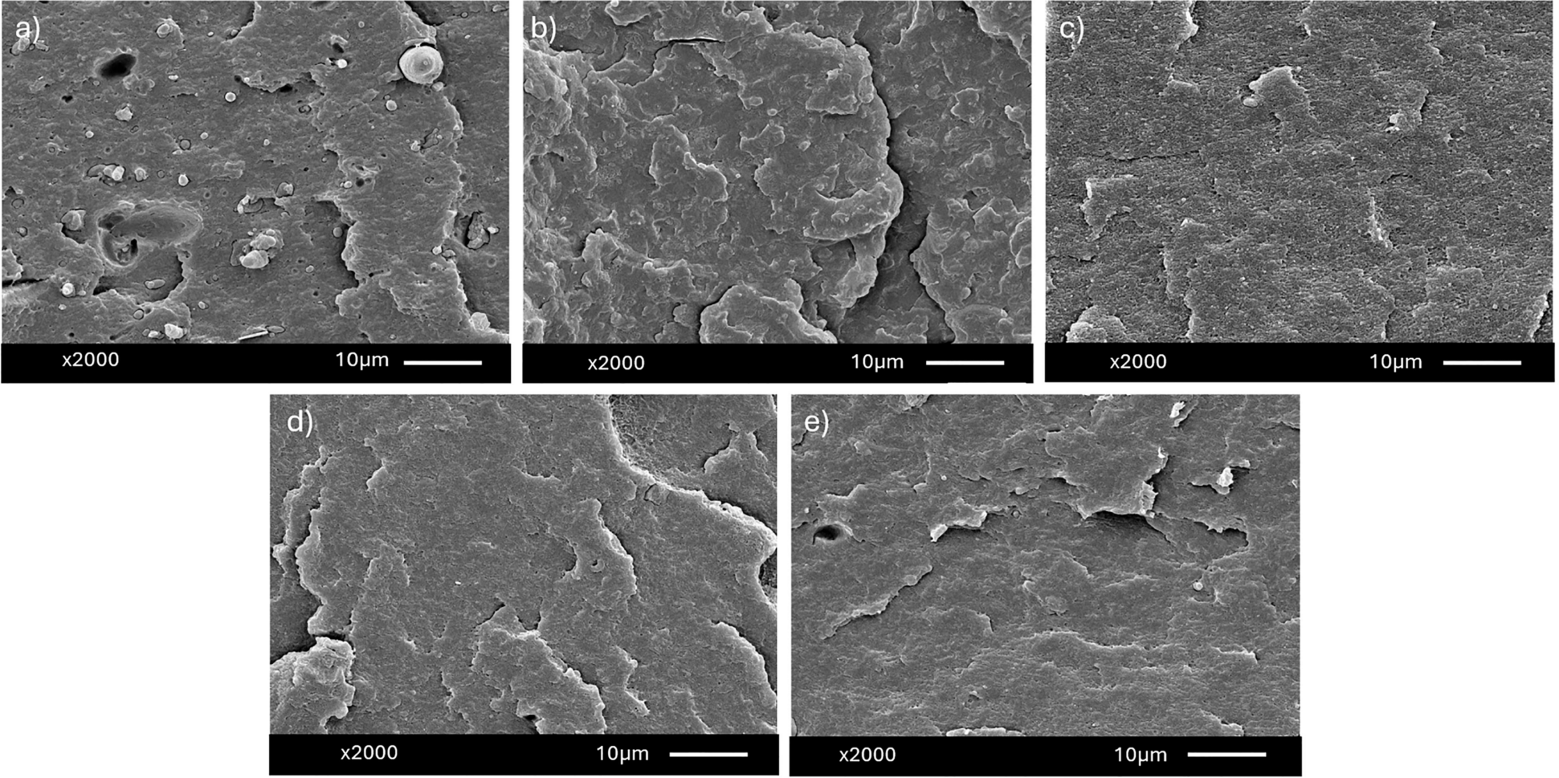
SEM images of m-POs samples. (a) 80PO, (b) PW 4K, (c) B52 4K, (d) 1 kg, and (e) 50 kg sample structure in SEM analysis.

The SEM analysis in Figure 8 shows the phase morphology and interfacial compatibility of the various m-POs blends. The uncompatibilized 80PO sample (Figure 8a) shows distinct phase separation between the wHDPE and rPP domains. This separation between both polymers shows weak interfacial adhesion, which is consistent with the reduced mechanical properties seen in this blend.^25,29,32^

The addition of paraffin wax (PW 4K, Figure 8b) and Sasol B52 (B52 4K, Figure 8c), both containing 4 wt % Kraton, produces a much smoother and uniform phase distribution. The Kraton compatibilizer works at the interface of wHDPE and rPP, increasing adhesion and decreasing interfacial tension. This leads to smaller, more evenly spread domains, showing improved phase compatibility. The smoother fracture surfaces in these samples indicate better toughness, which can be proved with the mechanical properties data from Figures 1, 4, and 6.^34,35^

The morphology of the 1 and 50 kg m-POs samples (Figure 8d,e) is reasonably uniform and consistent in phase distribution, but larger dispersed domains still exist, particularly in the 50 kg sample. Overall morphology suggests successful compatibilization, with rPP phases distributed quite evenly throughout the wHDPE matrix.^14,26,27^

## 4 Conclusions

We have successfully investigated the effect of wHDPE and rPP compositions in r-PO blends on these materials’ MFI and mechanical properties. Overall, the modulus of elasticity for r-PO blends increased with a higher rPP content, while elongation drastically decreased. The impact strength was significantly reduced in blends with increased rPP contents in the r-PO blends. When Sasol and paraffin wax were blended with the m-POs, there was a consistent increase in the MFI with the addition of either wax, but at 8%, paraffin wax offered a much larger increase compared to Sasol B52. Sasol often yielded slightly better performance in tensile strength, modulus of elasticity, and impact strength of the m-POs containing 4 wt % Kraton, while paraffin wax offered better performance in terms of elongation at break. Interestingly, at higher concentrations of Kraton (8 wt %), the difference between paraffin wax and Sasol-based blends became less noticeable. m-POs produced at a 50-kg scale yielded the closest properties in terms of tensile strength, elongation at break, and impact strength to that of virgin HDPE and exhibited significantly better elongation at break performance. The thermal properties support the conclusion that the combination of paraffin wax and Kraton did not significantly affect the thermal properties of the blends. SEM analysis reveals that homogeneity is observed at all scales, indicating that these formulations are suitable for industrial-scale recycling applications. This work demonstrates the successful use of paraffin wax as an RM in the presence of Kraton as a compatibilizer for the large-scale production of these r-PO blends to create virgin HDPE-like materials.

## Supporting Information Available

Mechanical properties and MFIs of various PO/PP blends; thermal properties for rPP, wHDPE, and m-POs samples (PDF)
