# Supplementary material for: Large-Scale Compatibilization of Postconsumer Polyolefins in the Presence of Paraffin Wax as a Rheology Modifier
Source: ACS Omega. 2025 Apr 11;10(15):15172–80. doi: 10.1021/acsomega.4c10910 (PMC12019720; doi:10.1021/acsomega.4c10910)
Supplement: Supplementary file 1 — ao4c10910_si_001.pdf [file ao4c10910_si_001.pdf]

Supporting Information

For

Large-Scale Compatibilization of Postconsumer Polyolefins in the Presence of Paraffin Wax as a Rheology Modifier

Anurag Ganapathi, Rishi Sharma, Mohamed A. Abdelwahab, Muhammad Rabnawaz*

School of Packaging, Michigan State University, East Lansing, MI 48824-1223, USA

***Corresponding Author:** Muhammad Rabnawaz, E-mail: rabnawaz@msu.edu

Table S1. Mechanical properties and MFIs of various PO/PP blends

Name	wHDPE	rPP	MFI (g/10 min)	Tensile Strength (MPa)	Elongation at Break in (%)	Modulus (MPa)	Impact Strength (kJ/m^2)	Break Type
**PP**		100	10.7 ± 1.5	23.3 ± 0.9	110± 26	590 ± 17	4.3 ± 0.3	Full
**HDPE**	100		0.6 ± 0.1	26.3 ± 1.2	235 ± 43	468 ± 14	69.4 ± 2.2	Unbroken
**80PO**	80	20	1.3 ± 0.1	22.2 ± 0.3	476 ± 12	526 ±90	18.9 ± 1.2	Hinge
**60PO**	60	40	2.2 ± 0.2	23.5 ± 0.7	449 ± 148	574 ± 37	4.3 ± 0.2	Full
**40PO**	40	60	5.4 ± 1.0	22.9 ± 0.3	10 ± 0.9	612 ± 30	3.7 ± 0.1	Full
**20PO**	20	80	12.5 ± 1.6	23.2 ± 0.1	10.1 ± 0.6	642 ± 10	3.5 ± 0.2	Full

Table S2. Thermal Properties for rPP, wHDPE and m-POs samples

Sample	T_c (°C)	T_{m1} (°C)	T_{m2} (°C)	ΔH_{m1} (J/g)	ΔH_{m2} (J/g)	X_{c1} (%)	X_{c2} (%)
rPP	123 ± 0.01	-	160 ± 0.1	-	87 ± 1	-	42 ± 1
wHDPE	114 ± 1.0	134 ± 1.3	-	177 ± 5.6	-	61 ± 2	-
80PO	117 ± 0.1	130 ± 0.1	162 ± 0.1	115 ± 6.4	8 ± 3	49 ± 3	20 ± 7
B52 4K	127 ± 0.2	129 ± 0.1	163 ± 0.2	39 ± 0.4	40 ± 1	16 ± 1	80 ± 3
B52 8K	126 ± 0.3	130 ± 0.4	163 ± 0.44	35 ± 5.4	39 ± 3	14 ± 2	79 ± 7
PW 4K	116± 0.1	131 ± 0.2	165 ± 0.23	116 ± 7.3	9 ± 2	47 ± 3	17 ± 3
PW 8K	126 ± 0.4	129 ± 0.1	163 ± 0.28	34.2 ± 0.1	40 ± 1	14 ± 1	80 ± 1
1 kg	114 ± 0.6	120 ± 1.0	163 ± 0.11	140 ± 0.6	8 ± 1	57 ± 1	16 ±2
50 kg	115 ± 1.4	132 ± 2.2	163 ± 0.58	131 ± 4.4	9 ± 1	53 ± 2	18 ± 1

*) The melting points are T_{m1} and T_{m2} for wHDPE and rPP, respectively; the glass transition temperature is T_g; the degree of crystallinity is X_{c1} and X_{c2} for wHDPE and rPP, respectively; the enthalpy of melting is ΔH_{m1} and ΔH_{m2} for wHDPE and rPP, respectively.
